# Impact of Pomegranate Extract Supplementation on Physical and Cognitive Function in Community-Dwelling Older Adults Aged 55–70 Years: A Randomised Double-Blind Clinical Trial

**DOI:** 10.3390/geriatrics10010029

**Published:** 2025-02-17

**Authors:** Grace Farhat, Jhama Malla, Emad A. S. Al-Dujaili, Jay Vadher, Pradeepa Nayak, Kenneth Drinkwater

**Affiliations:** 1 Faculty of Health and Education, Manchester Metropolitan University, Manchester M15 6BG, UK; p.nayak@mmu.ac.uk; 2 Faculty of Health and Medical Sciences, University of Surrey, Guildford GU2 7YH, UK; j.malla@surrey.ac.uk; 3 Centre for Cardiovascular Science, Faculty of Medicine and Veterinary Medicine, Queen’s Medical Research Centre, Edinburgh EH16 4TJ, UK; ealduja1@exseed.ed.ac.uk; 4 Faculty of Sport and Exercise, Manchester Metropolitan University, Manchester M15 6BH, UK; j.vadher@mmu.ac.uk; 5 Department of Psychology, Manchester Metropolitan University, Manchester M15 6GX, UK; k.drinkwater@mmu.ac.uk

**Keywords:** pomegranate extract, ageing, cognitive decline, cognitive function tests, physical function

## Abstract

**Background/objectives:** Cognitive decline and loss of physical function are common concerns in older adults, with limited effective interventions available. This study aimed to assess the impact of pomegranate extract (PE) supplementation on cognitive and physical function in older adults aged 55–70 years. **Methods**: A randomised, double-blind placebo-controlled trial was conducted with 86 participants, who were assigned to receive either PE (740 mg) or a placebo (maltodextrin) daily for 12 weeks. Cognitive function was assessed using computerised tests (Corsi, digit span, Wisconsin Card Sorting Test (WCST), Tower of Hanoi, Stroop test and Rey auditory verbal learning test). Physical function was measured through assessments of standing balance, gait speed, chair sit to stand and grip strength. **Results**: There was a significant effect of treatment and time on WCST performance (F (1,2) = 2.718, *p* = 0.05), while trends towards better outcomes in the PE group were noted for digit span, Tower of Hanoi and Stroop tests. Physical function did not seem to be affected by the intervention, but results may have been limited by the high baseline physical activity levels and full mobility of the older adults. **Conclusions:** This was the first study to examine the effect of PE on cognitive and physical function over a duration of 12 weeks. Findings suggest that PE supplementation has potential in improving cognitive function and may offer a promising approach to preventing cognitive decline in ageing adults. Further controlled and well-designed long-term studies are needed to establish the long-term effects of PE on cognitive and physical health, along with the mechanisms of action involved.

## 1. Introduction

Population ageing is a global phenomenon, with over 1 billion individuals currently aged 60 years and older [1]. One of the most significant challenges associated with ageing is the decline in physical and cognitive functions, which significantly impacts quality of life and daily functions [2]. Attention and memory are among the cognitive functions most significantly affected by ageing [3], alongside a decline in muscle strength, which contributes to reduced physical function [4]. Research suggests that physical and cognitive functions are closely interconnected: age-related functional decline is associated not only with reductions in physical abilities, such as balance and grip strength, but also with impairments in cognitive functions, including memory deficits and decreased executive functioning [5,6]. Preventing or slowing the decline in both physical and cognitive capacities is, therefore, a critical area of research to improve the well-being of older adults. With cognitive decline varying among individuals and potentially beginning as early as middle age [7], it is crucial to explore preventive strategies targeted at this age group.

Polyphenols, recognised for their antioxidant and anti-inflammatory properties have demonstrated potential in enhancing cognitive function in observational studies [8,9,10]. Among these, pomegranate stands out as one of the fruits with the highest antioxidant capacity [11]. Research on the role of pomegranate in cognitive function has been encouraging but remains relatively limited. A substantial number of preclinical trials have reported a beneficial role of pomegranate on cognitive function. A systematic review, which analysed 20 animal studies and 4 human randomised controlled trials, found that pomegranate was associated with improvements in specific cognitive domains. These effects are primarily attributed to the antioxidant and anti-inflammatory properties of pomegranate compounds, particularly punicalagin and ellagic acid, which may reduce oxidative stress and neuroinflammation, both critical factors in cognitive decline [12]. Findings from a one-year randomised controlled trial showed that the regular consumption of pomegranate juice improves verbal memory in middle-aged and older adults [13]. While these findings need to be replicated, more sustainable and cost-effective alternatives such as pomegranate extract must be tested to determine if they provide comparable benefits. Acute ingestion of pomegranate extract has previously shown improvements in working memory and logical reasoning in healthy adults [14], which warrants further investigation.

As for physical function, pomegranate extract has demonstrated potential in improving physical function in older adults and aiding muscle recovery in athletes [15,16], yet studies have involved small sample sizes and been short-term, highlighting the need for further exploration of this effect. This study, therefore, aimed to investigate the effects of pomegranate extract on physical and cognitive function in adults aged 55–70 years, using a battery of standardised cognitive and physical function tests. Results could provide valuable insights for future longitudinal studies examining the impact of pomegranate extract on cognitive health, physical performance and the prevention of age-related functional decline.

## 2. Methods

The trial was granted ethical approval by the Manchester Metropolitan University Faculty of Health and Education (reference number: 47627, 28 November 2022) and was registered with clinicaltrials.gov (NCT05588479, 18 October 2022). Participants signed an informed consent before taking part.

### 2.1. Study Design, Participants and Procedure

We conducted a two-arm double-blind parallel trial where participants were randomly allocated to receive either placebo capsules (maltodextrin) or pomegranate extract (PE) capsules (740 mg of PE) daily for 12 weeks. Participants were not restricted in terms of the timing of capsule consumption, as long as they adhered to the daily dosage. This dosage was selected because it falls within the range of 500–1000 mg, shown in prior studies to reduce serum inflammatory markers (the study’s primary outcome) [17]. Furthermore, our previous research demonstrated the acute cognitive benefits of the same extract at a dose of 700 mg [14]. PE was specifically prepared for the study and produced by fully automated hydro-extraction technology using whole pomegranate fruit growing in Spain. The composition of each PE capsule included 36% punicalagins and 1.3% ellagic acid, as determined by the HPLC method using an Applied Biosystems Model 757 absorbance detector. The pomegranate and placebo capsules were identical in appearance and were distributed to participants in two batches: one at baseline and the other at week 6. Allocation was conducted by recruitment staff using a computer-generated randomisation method. Both intervention and placebo groups were matched for age, gender and BMI.

Individuals of all genders aged 55–70 years, with no history of chronic diseases (e.g., heart disease, diabetes, renal disease or liver disease) and classified as having a normal weight (BMI 18.5–24.9 kg/m^2^) or being overweight (BMI 25–29.9 kg/m^2^) were eligible for the study. Participants attended the Physiology Laboratory at baseline, week 6 and week 12. During each visit, anthropometric measurements were recorded, including weight (using a Marsden DP2400 digital scale), height (measured with a Seca^®^ 711 stadiometer, Seca GmbH & co. KG., Hamburg, Germany), waist and hip circumferences (measured with an elastic tape) and body composition (assessed using air displacement plethysmography with the BodPod^®^ GS-X: Cosmed, Rome, Italy). These measurements were then followed by computerised cognitive assessments and physical function tests.

Participants were asked to maintain their diet and physical activity for the whole intervention period. To monitor dietary intake, they were asked to fill a 3-day diet diary at baseline, week 6 and week 12. They also completed a paper-based socio-demographic questionnaire before or during their first visit, which included details on age, gender, occupation and physical activity habits. They were given a paper-based diary to log the dates of capsule intake and were asked about their adherence to capsule intake at the second and third appointments.

### 2.2. Computerised Cognitive Tests

Participants were seated in front of a computer and provided with brief instructions on how to start. They completed six standardised and validated cognitive tests, with written, specific instructions given before each test. The tests used were developed using normative data from a representative sample of the population, typically from healthy participants without cognitive impairments. The entire testing session was expected to take between 30–45 min, depending on each participant’s performance.

Corsi Block-Tapping Test: designed to assess short-term working memory and sequencing. It is based on the ability of individuals to replicate a sequence of taps displayed on the computer screen. The sequence began with two blocks and gradually increased in complexity, becoming more challenging as the participant’s performance allowed. The task continued until the participant was no longer able to accurately repeat the sequence. Individuals with healthy brain function normally have a Corsi block span of 5–7 blocks [18].

Digit span test: measures verbal short-term memory and working memory. Participants were presented with a sequence of two digits and were required to repeat the sequence accurately. If successful, a longer sequence was then presented. This process continued, with the sequence length increasing each time until the participant was unable to correctly recall the sequence. The longest sequence that the participant could accurately repeat was considered their digit span [19]. It is suggested that an average individual can recall seven digits [20].

WCST: aims to measure cognitive reasoning. Participants were required to classify cards based on changing criteria (colour, shape or number of symbols). The classification rule changed every 10 cards, and participants were expected to adapt to these changes. Performance was assessed by tracking total errors, which included the sum of perseveration errors (continuing to apply the old rule) and non-perseveration errors [21].

Stroop test: measures a person’s ability to inhibit automatic responses and manage cognitive interference, reflecting their capacity to suppress habitual reactions in favour of less typical ones. In this task, participants were asked to identify the ink colour of written colour names while ignoring the word itself (e.g., the word “blue” written in red ink). The Stroop effect represents the difference in average response time between incompatible trials and compatible trials [22].

Tower of Hanoi: measures planning, problem-solving and executive function abilities. In this task, participants were presented with three pegs and a set of discs of varying sizes and were asked to move all the discs from the starting peg to a target peg, following two main rules: only one disc could be moved at a time, and a larger disc could not be placed on top of a smaller one [23].

Rey auditory verbal learning test: a neuropsychological assessment used to evaluate memory function. Participants were verbally presented with a list of 15 words (List A) over five trials. After each presentation, they were instructed to recall as many words as possible by writing them down. Following these initial trials, participants completed other cognitive tasks (e.g., Tower of Hanoi, WCST, Stroop test, digit span test and Corsi tests). They were subsequently introduced to a new list of words (List B) and were asked to recall as many words as possible over five trials (Trial 6). Participants were then asked to recall as many words as possible from the original List A (referred to as the Rey verbal original recall test) This test was adapted from Bowler (2021) [24].

### 2.3. Physical Function Tests

Strength was measured using handgrip strength. For this test, participants were asked to hold a hand-held dynamometer above their head and lower their arm to their side whilst squeezing as hard as possible. Participants completed two repetitions on each arm with the average strength value (in kg) used in the analysis.

Balance and functional mobility were assessed using standing balance, gait speed and chair sit to stands, and protocol was adapted from Puthoff (2008) [25]. For the standing balance assessment, participants were first asked to stand unsupported for 10 s with their feet placed side-by-side. If they successfully completed this, they progressed to standing with the heel of one foot positioned beside the great toe of the other foot for 10 s. Next, participants were instructed to stand with one foot placed directly in front of the other for 10 s. Finally, if successful, they were asked to stand on one foot for the same duration. The percentage of people completing the full standing balance test was then assessed.

Gait speed is a widely used method for assessing walking performance in older adults, providing a quick, safe and reliable measure [25]. It was evaluated over a 10 m walkway, marked by cones or tape at each end. Participants were instructed to walk at their usual pace between the markers. The test was conducted twice, and the fastest time was recorded.

For the chair sit-to-stand test, participants sat on a chair with their arms crossed over their chest and feet flat on the floor. Upon instruction, they stood up while keeping their hands in place. If a participant was unable to stand from this position, the test was discontinued. Otherwise, they performed five sit-to-stand repetitions as quickly as possible, and the time taken to complete these movements was recorded.

### 2.4. Power Calculation and Statistical Analysis

The sample size was evaluated based on the primary outcome of the study (Interleukin-6 levels) which generated a sample size of 84 participants. However, we determined that a sample size of 38 participants per arm was sufficient to detect a 10% difference in word recall between the groups, given an alpha level of 0.05 and power of 0.80. Results were derived from the study of Wood et al. (2023) [26].

Data were analysed using SPSS version 29 (IBM, Chicago, IL, USA) and presented as mean (SD), unless otherwise noted as standard error (SE). Normality was assessed using the Kolmogorov–Smirnov test. For non-parametric data, variables were logarithmically transformed before analysis. Baseline characteristics were analysed using descriptive statistics and frequencies. The independent t-test and Chi-square exact test were used to assess between-group differences in baseline characteristics for continuous and categorical variables, respectively. Physical activity levels were converted into MET (metabolic equivalent of task) values, which represented the energy expenditure for each activity compared to resting. MET values were estimated based on commonly available guidelines for various activities [27]. The interactions between treatments (PE vs. PL) and time (baseline, week 6 and week 12) were evaluated using linear mixed models. The impacts of occupation and physical activity levels on the outcomes were assessed using linear mixed-model effects repeated with the variables included as fixed factors or covariates, respectively. For significant differences, pairwise comparisons were conducted using the Bonferroni correction. Statistical significance was set at *p* ≤ 0.05.

## 3. Results

**Figure 1:**
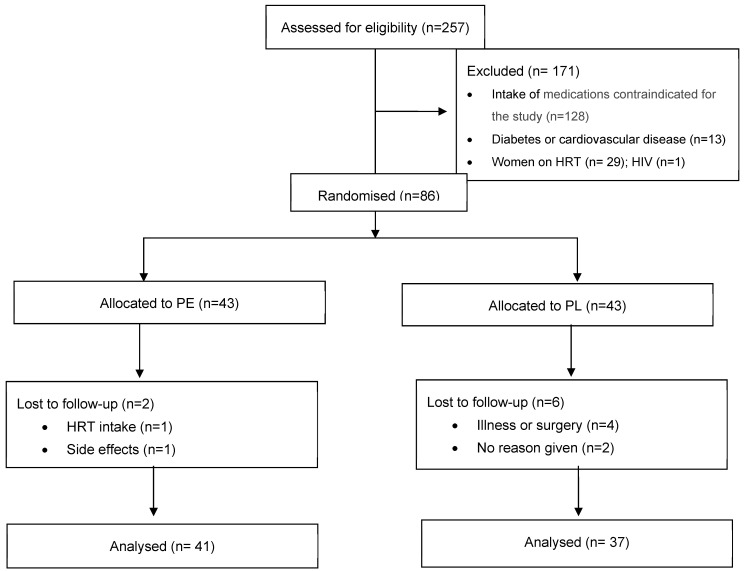
Consort flow diagram. Abbreviations: PE: pomegranate extract; PL: placebo; HRT: hormone replacement therapy.

The study advert attracted 355 responses, with 257 individuals progressing to screening. Of these, 86 were enrolled in the study; 76 participants completed it, while 2 participants only completed two time points. The CONSORT flow diagram is shown in Figure 1.

Participants were predominantly females (61.54%), with a mean age of 61.28 (4.37) years and with most holding professional or managerial positions (57.69%). They were, overall, physically active (751 (132) MET-minutes/week), with 76% engaging in more than one activity per week, and only two participants reporting no physical activity. Baseline characteristics of the study population are presented in Table 1.

### 3.1. Effect of Pomegranate Extract on Cognitive Function Assessed Through Cognitive Tests

#### 3.1.1. Cognitive Reasoning: WCST Results

**Figure 2:**
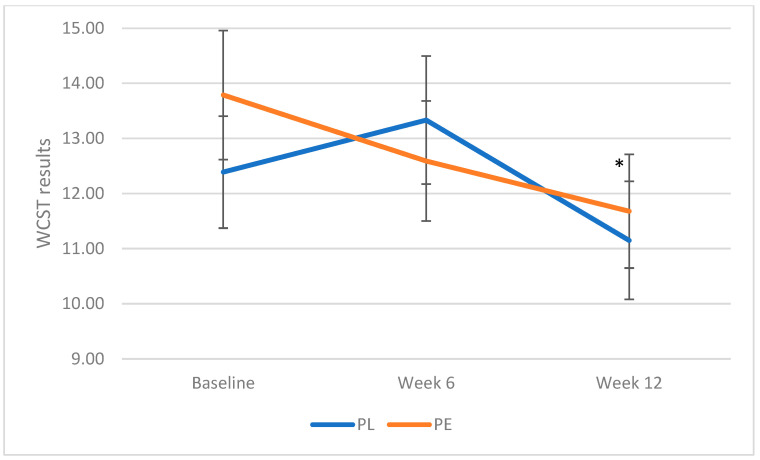
Effect of pomegranate extract on WCST performance. Data were analysed via linear mixed models following logarithmic transformation of data (non-parametric data). Data are expressed as means (SE). * *p* ≤ 0.05, significant decrease from baseline. Abbreviations: PE: pomegranate extract; PL: placebo; WCST: Wisconsin Card Sorting Test.

No significant difference in baseline values of WCST was noted between the two groups (*p* = 0.33). Results showed a significant effect of treatment and time on WCST performance (F (1,2) = 2.718, *p* = 0.05), with the numbers of errors decreasing more significantly at week 12 in the PE group compared with the PL group (Figure 2). This decrease was primarily due to a reduction in non-perseveration errors count (F (1,2) = 3.06, *p* = 0.05).

#### 3.1.2. Effects of PE on Short-Term Memory Performance

**Figure 3:**
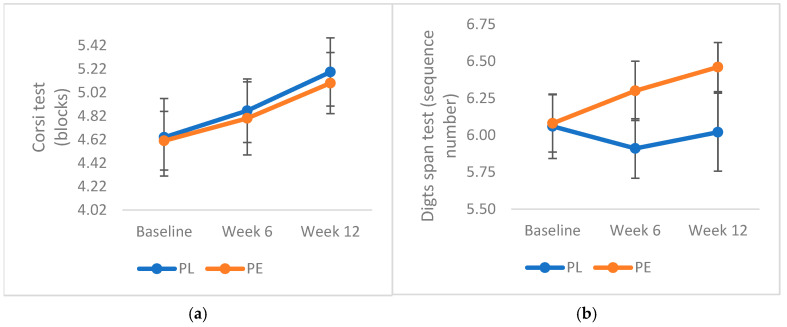

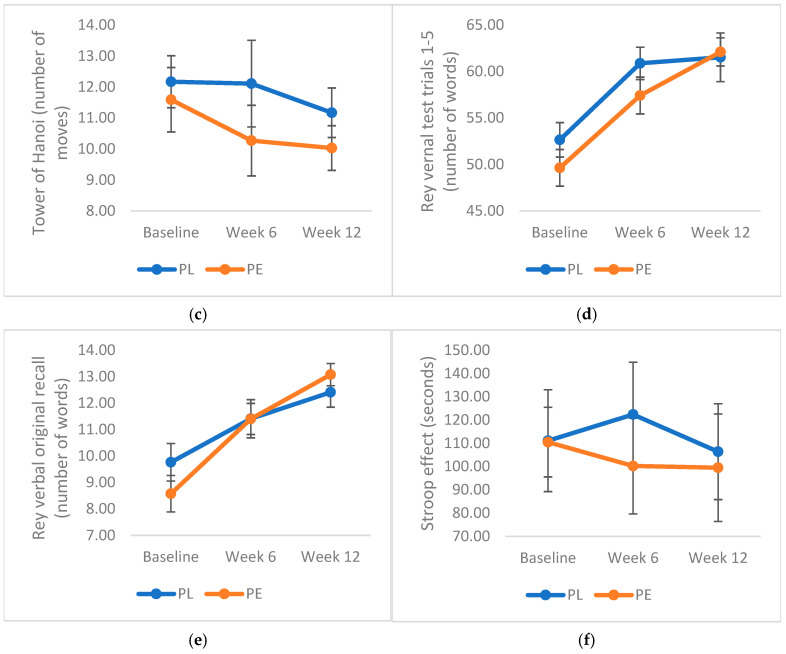
Effects of pomegranate extract on different cognitive test results. (**a**) Corsi test; (**b**) Digits span; (**c**) Tower of Hanoi; (**d**) Rey verbal test trials 1-5; (**e**) Rey verbal original recall & (**f**) Stroop effect. Abbreviations: PE: pomegranate extract; PL: placebo. Data were analysed via linear mixed models following logarithmic transformation of data (non-parametric data). Data are expressed as means (SE). No significant effects of treatment and time on the results were noted (*p* > 0.05).

Corsi Block-Tapping test: At baseline, participants achieved an average score of 4.62 (SD 1.79) blocks; thus, only 62.3% of participants in this study exceeded the threshold of 5 blocks. Mixed model analysis revealed no significant impact of PE on Corsi block scores (F (1,2) = 0.97, *p* = 0.38); both groups displayed a trend of learning effects throughout the intervention (Figure 3a).

Digit span test: The mean digit span at baseline for all participants was 6.07 (1.24) digits. Analysis revealed a trend towards a more consistent increase in the number of digits in the longest remembered sequence in the PE group compared with the PL group. However, this increase was not statistically significant (F (1,2) = 0.3, p = 0.74) (Figure 3b).

Tower of Hanoi test: Similar outcomes were noted, with a trend towards a lower number of moves in the pomegranate group at week 6 compared with the placebo group after 12 weeks (F (1,2) = 0.06, p = 0.94) (Figure 3c).

Rey auditory verbal learning test: Analysis revealed no significant effect of treatment on the average scores from Trials 1 to 5 (F (1,2) = 0.96, *p* = 0.39), but a significant learning effect was observed, as scores progressively improved across visits in both groups (Figure 3d). Similarly, no significant effect of treatment and time was found for Trial 6 (F (1,2) = 1.84, *p* = 0.16) or the Rey verbal original recall test (F (1,2) = 2.16, *p* = 0.12 (Figure 3e).

### 3.2. Measurement of Cognitive Inhibition: Stroop Test

An analysis of the Stroop test results revealed a clear and consistent trend of decreasing response times over time in the PE group, without reaching statistical significance (F (1,2) = 0.53, *p* = 0.59) (Figure 3f).

### 3.3. Effect of Pomegranate Extract on Physical Function Tests

Analysis via linear-mixed model effects revealed that in fully mobile older adults, PE did not seem to improve physical function (Table 2). Overall, 97.4% of participants successfully completed the standing balance test at both baseline and week 6, while 96.1% completed it at week 12, with participants evenly distributed across both PE and PL groups.

### 3.4. Impact of Multiple Variables on the Outcomes

A linear mixed-model analysis of the effect of treatment and time on cognitive function, using occupation as a fixed factor revealed no significant effect of occupation on any of the cognitive tests (*p* > 0.05). No significant impact of physical activity levels (MET) was observed on either physical or cognitive function tests (*p* > 0.05).

### 3.5. Compliance

Participant reported a good compliance, which was estimated to be around 87%. Only two participants reported an upset stomach during the intervention (PL group). A random analysis of diet diaries of 30 participants showed no significant changes in energy intake throughout the intervention (*p* = 0.31 for PL group and *p* = 0.24 for PE group). Additionally, no significant differences in macronutrient intake (protein, fat and carbohydrate) were noted (*p* > 0.05). Physical activity levels did not significantly change throughout the intervention (*p* = 0.67 or PL group and *p* = 0.28 for PE group).

## 4. Discussion

This trial investigated the impact of 12 weeks of daily PE supplementation on improving physical and cognitive function, as well as its potential to enhance well-being in older adults aged 55–70 years. The trial is timely, given the gaps in the literature regarding which foods, dietary patterns or bioactive components can effectively support healthy ageing and brain ageing. Despite increasing evidence suggesting that diet is a modifiable factor for promoting healthy ageing, more research is needed in this area [28].

Outcomes demonstrated a significant improvement in cognitive reasoning in the PE group, with notable trends towards enhanced short-term and working memory as well as an improved ability to limit cognitive interference. Following pomegranate supplementation, significant improvements in cognitive function, including memory and executive function, have been reported in healthy individuals [14]; in patients recovering from stroke [29] or heart surgery [30]; in those with mild cognitive impairment [29] and in older adults with memory complaints [31]. However, these studies were short-term and involved small sample sizes, limiting generalisability. Notably, a study with a larger cohort found that one year of pomegranate juice supplementation significantly enhanced visual memory in adults [13]. The trends observed in our study related to improvements in short-term memory and cognitive function, although not statistically significant, are noteworthy and suggest that PE may offer broader cognitive benefits that could become more pronounced with a longer intervention period. Additionally, a larger sample size would have provided the opportunity to detect smaller effect sizes.

Given that shorter-term studies have reported significant cognitive improvements, it is possible that the duration of our intervention was neither short enough to capture early effects nor long enough to detect more substantial benefits. Moreover, it is possible that we missed capturing early effects, as our outcome assessments did not specifically target short-term changes. It is worth noting that the participants’ high levels of motivation, and the fact that many held professional or managerial positions may not have been representative of the general population. Future research should aim to determine the optimal duration of supplementation for improving cognitive outcomes, emphasizing the inclusion of individuals from diverse socio-economic backgrounds to better assess the preventive effects of PE.

Although the exact mechanism is not yet clear, it is known that urolithins, metabolites of ellagitannins which are produced by gut bacteria from pomegranate polyphenols, can cross the blood–brain barrier and positively impact cognitive function [32]. Additionally, punicalagins have been reported to improve memory and learning in ageing mice, with evidence suggesting enhanced cognitive function and protection against neuroinflammation [33]. Similarly, ellagic acid has been shown to improve memory and reduce cognitive impairments associated with neurodegenerative diseases [34,35]. These studies suggest that the reduction in inflammation and oxidative stress may be primary mechanisms underlying their cognitive benefits. However, further research is needed to confirm their efficacy in clinical settings, and the specific mechanisms of action on cognitive function warrant further investigation.

The lack of significant improvement in physical function observed in this study may indicate that PE supplementation provides limited additional benefits for physically active and independent older adults. Moreover, the physical tests used in our study are normally used to assess basic motor function. Consequently, they are not sufficiently challenging to detect improvements in participants who can already perform these tasks with ease. Baseline physical status and more advanced assessments are needed to accurately measure the potential effects of PE on physical function.

Our results suggest that pomegranate extract may have potential in improving cognitive function and preventing cognitive decline. A large, well-powered study with a long-term intervention and multiple assessment points (e.g., from 2 weeks to 12 months), and including participants from diverse educational backgrounds is needed to establish such outcomes. Most importantly, standardising the dosage of polyphenols is key to facilitating comparison between studies and providing appropriate advice to the public. Future studies could also benefit from using other, more objective measures of cognitive function, such as fMRI (functional magnetic resonance imaging).

This study had several strengths. To our knowledge, it was the first to examine the effect of PE on cognitive and physical function over a duration of 12 weeks. The study was well-powered and used multiple standardised cognitive and physical tests to enhance reliability. However, the duration of the intervention may not have been sufficient to capture the full impact of pomegranate extract on cognitive function. Additionally, the study’s eligibility criteria, which included only individuals without diseases and those who were relatively healthy, may have primarily attracted physically active individuals in professional or managerial positions. This limitation could impact the generalisability of the results. Moreover, the battery of physical tests may not have been challenging enough to detect changes beyond basic physical function, and the multiple cognitive assessments may have introduced a testing burden on participants. Furthermore, while educational and occupational levels are often interlinked, obtaining information about education could have been better controlled for its impact on the intervention. Lastly, including a quality-of-life questionnaire could have explored whether the intervention had short-term impacts on participants’ overall well-being, providing valuable directions for future research.

In conclusion, this study provides evidence that PE supplementation may have potential cognitive benefits for older adults in the areas of cognitive reasoning as well as memory, executive function and cognitive interference, suggesting the need to explore these effects with longer intervention periods. While the lack of significant changes in physical function may indicate limited benefits for physically active and independent individuals, PE may still hold promise for those experiencing physical decline, which could be explored. Future research with larger sample sizes and longer follow up are needed, along with exploring the underlying mechanisms of action, to help support healthy ageing and the maintenance of independence in older adults.

## Figures and Tables

**Table 1:** Characteristics of study participants.

Characteristics	All Participants (*n* = 78)	PE Group (*n* = 41)	PL Group (*n* = 37)
Age (mean (SD) years) Gender (n)	61.28 (4.37)	61.81 (4.59)	60.8 (4.15)
Female	48	25	23
Male	30	16	14
Occupation (n)			
Professional and managerial occupations	45	21	20
Associate professional and technical occupations	5	2	3
Administrative, sales and trade occupations	8	8	4
Retired/semi-retired/unemployed	18	10	10
Physical activity (MET-minutes/week)	751 (132)	742 (153)	747 (161)
Smoking status (n) Smoker Non-smoker	3 75	1 40	2 35
BMI (kg/m^2^)	23.99 (3.23)	23.90 (3.14)	24.10 (3.32)
Waist circumference (cm)	84.88 (10.17)	83.86 (9.94)	86.02 (10.43)
Waist-to-hip ratio	0.84 (0.07)	0.84 (0.07)	0.85 (0.06)
Body fat percentage (%)	22.32 (10.16)	22.49 (9.49)	22.14 (10.99)

Data are reported as mean (SD). Baseline group comparisons were performed using independent *t*-tests for continuous variables and chi square exact test for categorical variables. No significant differences were found between the groups (*p* > 0.05). Abbreviations: PE: pomegranate extract; PL: placebo; MET: metabolic equivalent of task.

**Table 2:** Effect of pomegranate extract on physical function test outcomes. Data were analysed using linear mixed models. Abbreviations: PE: pomegranate extract; PL: placebo.

		Baseline		Week 6		Week 12	
		Mean (SD)	N	Mean (SD)	N	Mean (SD)	N
Right handgrip strength (Kg)	PL	27.22 (9.50)	37	27.52 (9.61)	37	28.0 (10.18)	36
	PE	27.81 (9.08)	41	28.92 (9.45)	41	28.10 (8.81)	40
Left handgrip strength (Kg)	PL	26.09 (10.43)	37	26.27 (9.96)	37	26.01 (8.49)	36
	PE	27.52 (9.79)	41	27.71 (10.18)	41	26.84 (9.39)	40
Gait speed (seconds)	PL	6.61 (0.88)	36	6.49 (0.65)	37	6.63 (0.8)	36
	PE	6.60 (0.85)	41	6.49 (0.65)	41	6.47 (0.82)	39
Chair to stand (seconds)	PL	11.12 (1.94)	36	10.70 (2.13)	37	10.35 (1.98)	35
	PE	10.24 (2.07)	41	10.66 (1.71)	41	10.30 (1.97)	39

Data were analysed via linear mixed models. Data are expressed as means (SD). No significant differences were noted (*p* > 0.05).

## Data Availability

Data described in the manuscript will not be made available due to being required for ongoing research and analysis.
