# Correction: Farhat et al. Impact of Pomegranate Extract Supplementation on Physical and Cognitive Function in Community-Dwelling Older Adults Aged 55–70 Years: A Randomised Double-Blind Clinical Trial. *Geriatrics* 2025, *10*, 29

**DOI:** 10.3390/geriatrics10060159

**Published:** 2025-12-04

**Authors:** Grace Farhat, Jhama Malla, Emad A. S. Al-Dujaili, Jay Vadher, Pradeepa Nayak, Kenneth Drinkwater

**Affiliations:** 1 Faculty of Health and Education, Manchester Metropolitan University, Manchester M15 6BG, UK; 2 Faculty of Health and Medical Sciences, University of Surrey, Guildford GU2 7YH, UK; 3 Centre for Cardiovascular Science, Faculty of Medicine and Veterinary Medicine, Queen’s Medical Research Centre, Edinburgh EH16 4TJ, UK; ealduja1@exseed.ed.ac.uk; 4 Faculty of Sport and Exercise, Manchester Metropolitan University, Manchester M15 6BH, UK; 5 Department of Psychology, Manchester Metropolitan University, Manchester M15 6GX, UK

There was an error in the original publication [1]. The percentage content of punicalagins was incorrectly specified in the manuscript due to an editing mistake, which has changed now from 75% to 36%.

A correction has been made to Section Methods, Section 2.1. Study Design, Participants and Procedure, Paragraph 1. The corrected paragraph is inserted below:

“The composition of each PE capsule included 36% punicalagins and 1.3% ellagic acid, as determined by the HPLC method using an Applied Biosystems Model 757 absorbance detector.”

The authors state that the scientific conclusions are unaffected. This correction was approved by the Academic Editor. The original publication has also been updated.
